# Supplementary material for: The interactome of intact mitochondria by cross-linking mass spectrometry provides evidence for coexisting respiratory supercomplexes
Source: Mol Cell Proteomics. 2017 Dec 8;17(2):216–32. doi: 10.1074/mcp.RA117.000470 (PMC5795388; doi:10.1074/mcp.RA117.000470)
Supplement: Supplemental Data [file supp_RA117.000470_133922_0_supp_23978_fzffwf.zip › spectra_annotation/mito_DR_spectra_annotation/354-1-9-1-88-1.pdf]

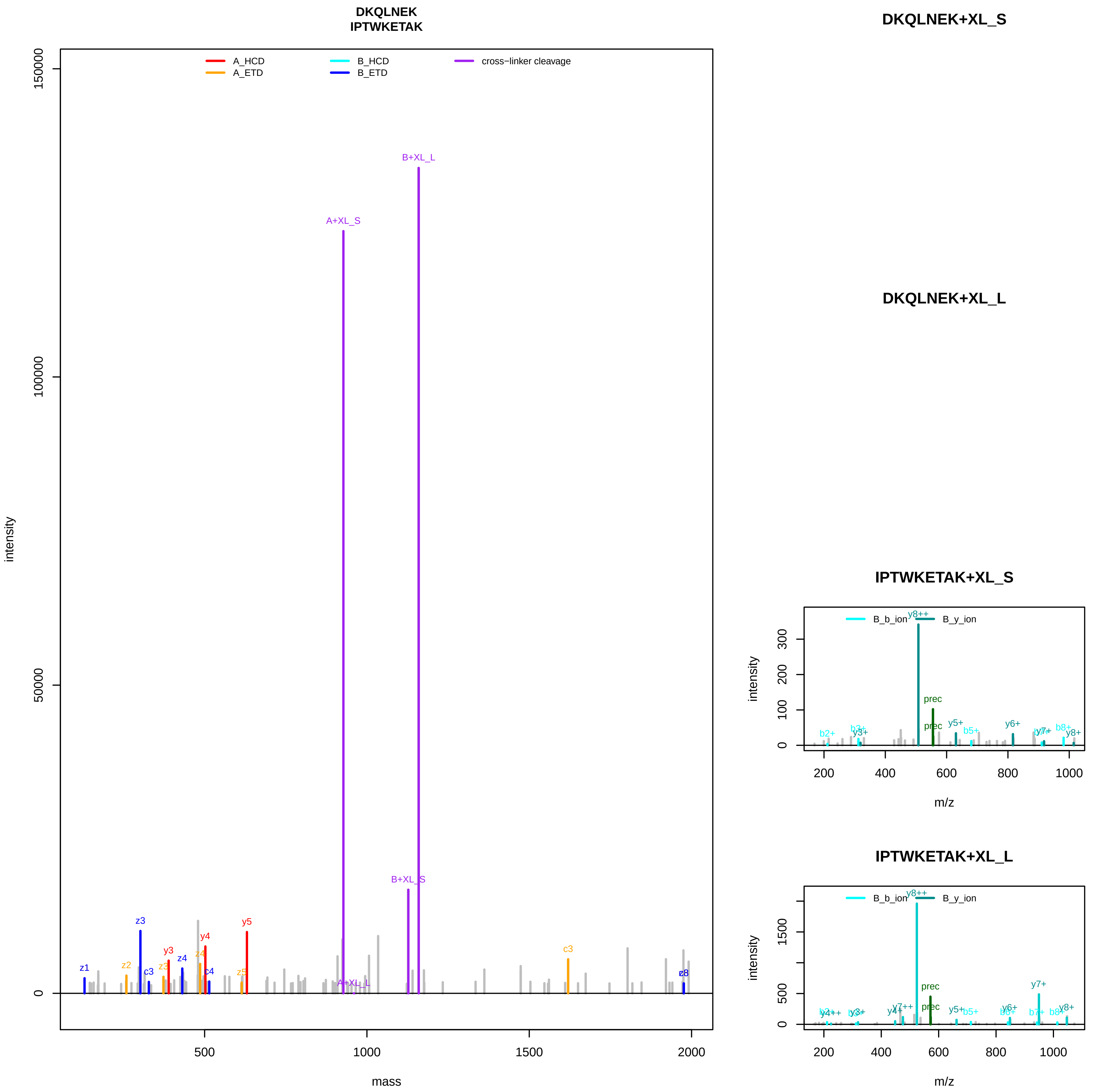
DKQLNEK
IPTWKETAK
A_HCD
A_ETD
B_HCD
B_ETD
cross−linker cleavage
intensity
mass
B+XL_L
A+XL_S
B+XL_S
A+XL_L
z1
z2
z3
c3
y3
z3
z4
y4
z4
c4
z5
y5
c3
c8
DKQLNEK+XL_S
DKQLNEK+XL_L
IPTWKETAK+XL_S
B_b_ion
B_y_ion
y8++
prec
y5+
b5+
y6+
y7+
b8+
y8+
b2+
b3+
y3+
m/z
IPTWKETAK+XL_L
B_b_ion
B_y_ion
y8++
prec
y7+
y7++
y4+
y3+
y4++
y5+
b5+
y6+
b6+
b7+
b8+
y8+
m/z
